# The impact of sex on HIV immunopathogenesis and therapeutic interventions

**DOI:** 10.1172/JCI180075

**Published:** 2024-09-17

**Authors:** Erin Mihealsick, Anna Word, Eileen P. Scully

**Affiliations:** 1 Graduate Program in Immunology and; 2 Division of Infectious Diseases, Department of Medicine, Johns Hopkins University School of Medicine, Baltimore, Maryland, USA.

## Abstract

Globally, the majority of people living with HIV are women or girls, but they have been a minority of participants in clinical trials and observational studies of HIV. Despite this underrepresentation, differences in the pathogenesis of HIV have been observed between men and women, with contributions from both gender- and sex-based factors. These include differences in the risk of HIV acquisition, in viral load set point and immune activation in responses to viremia, and differences in HIV reservoir maintenance. These differences obligate adequate study in both males and females in order to optimize treatments, but also provide a powerful leverage point for delineating the mechanisms of HIV pathogenesis. The shifts in exposure to sex steroid hormones across a lifespan introduce additional complexity, which again can be used to focus on either genetic or hormonal influences as the driver of an outcome. In this Review, we discuss consistent and reproducible differences by sex across the spectrum of HIV, from acquisition through pathogenesis, treatment, and cure, and explore potential mechanisms and gaps in knowledge.

## Overview

The HIV pandemic has claimed more than 40 million lives and has been a galvanizing force in research into the prevention, pathogenesis, and treatment of infectious diseases. It has also brought into sharp relief the tremendous variation in the effect of HIV infection when considered across a broad population, ranging from elite control (1, 2) to rapid disease progression (3). Sex and gender are linked to distinct risks of HIV acquisition, pathogenesis, and reservoir maintenance, concordant with the impact of sex on a variety of infectious and inflammatory conditions (4–6). Gender differences in health-associated behavior, access to care and resources, and social stressors have a profound role in health outcomes (7). Although this Review discusses HIV infection and outcomes through the lens of biological sex, particularly genetic and hormonal differences, all studies must be considered within the context of potential gender-based confounders and effects. Approaches to considering risk and research in a gender framework have been expertly discussed elsewhere (8, 9). We focus on differences by sex in the context of HIV, noting where gender factors may intervene, but seeking to leverage sex to identify mechanisms of pathogenesis and potential points for therapeutic intervention. Throughout the Review, we discuss studies of people living with HIV (PLWH) and, when discussing characteristics related to genetic (e.g., XX versus XY) and anatomic features (reproductive organs), specifically use the terms female and male. We have used the terms women and men to refer to cisgender individuals unless otherwise indicated and in describing data from studies in which these terms were used to describe the participants.

Herein, we indicate gaps and opportunities in the data and attempt to highlight comparisons where there are major confounders. A primary consideration is whether studies have adequate inclusion across sex and gender for valid conclusions. The initial description of AIDS as an acquired immune deficiency syndrome among men who have sex with men (10, 11) reflected the epidemic in the US and Europe, which has been dominated by men. This contrasts with the global epidemic, in which women account for 53% of PLWH (12). In sub-Saharan Africa, women constitute more than 61% of PLWH and 62% of new HIV diagnoses in this region (12) (Table 1). Distribution of the infection has important implications for the available data: a high proportion of biomedical research and funding originate in regions with male-dominated epidemics, contributing to an underrepresentation of female study participants (13–15). Elucidating immunologic mechanisms of phenotypic differences by sex across the spectrum from HIV acquisition through efforts towards a cure may facilitate the development of interventions that will serve all PLWH.

## Acquisition

Due to the very early integration of HIV into the host cell genome, viral eradication presents a formidable challenge, and a preventive vaccine remains crucial to ending the epidemic. In this section, we explore sex-specific features of HIV acquisition, including through vertical transmission, that inform the development of protective vaccines and deployment of preventive strategies including pre-exposure prophylaxis.

### Anatomic risks for HIV acquisition.

HIV can be acquired via parenteral exposure to blood products or through sexual activity. Although we lack significant data about sex-differential risk related to parenteral exposures, as discussed below, there is emerging data in the context of vertical transmission.

There are clear distinctions in acquisition risk thresholds based on sexual transmission. Broadly, the risk of sexual acquisition is dictated by the availability of cells that can be infected. This, in turn, is determined by anatomy and the levels of local inflammation (16). Receptive anal intercourse has the highest risk of transmission (17), with studies suggesting that the density of CD4^+^ T cells and inflammatory environment conditioned by the microbiome may contribute to elevating risk of acquisition, and that this risk may change with age (18). Penile-vaginal intercourse is associated with a higher risk of transmission to the female partner in high-income countries, with a more modest difference in other settings, notably where male circumcision is less common (19). Langerhans cells and CD4^+^ T cells present in the vaginal mucosa and penile foreskin are the primary targets for early HIV-1 infection (16, 20, 21). Medical male circumcision has been shown to significantly decrease the risk of HIV acquisition, likely both by reducing the local target cell populations and by eliminating inflammatory components of the foreskin microbiome (22). For females, there has been significant debate about the effect of hormone exposure on the vaginal mucosal environment. Two meta-analyses reported an approximately 40% increase in risk of HIV acquisition associated with use of depot medroxyprogesterone acetate (DMPA, a contraceptive injection under the brand name Depo-Provera) (23, 24). Suggested mechanisms include alterations in the epithelial layer, change in frequency of target cells, and inflammation and alterations in the microbiome. However, this is not supported by the results of a prospective randomized trial comparing DMPA with other contraceptive methods, in which there was not a substantial increase in risk (25). Importantly, this study also highlights that hormone exposure must be considered in context of the alternatives, which here would be alternative contraceptive methods or a pregnancy with the associated maternal risks (26). Nevertheless, a greater understanding of the influence of hormones on the local environment may provide information on factors that favor transmission. Further exploration of the effect of exogenous sex steroid hormone exposure in transgender individuals will also be important in order to optimize and target prevention efforts in this population (27).

In parallel with the risk for HIV acquisition that is conferred by the local availability of targets at a mucosal site is the potential protection conferred by vaccine-induced HIV-specific immune responses at these mucosal sites. With respect to humoral immunity, a meta-analysis of mucosal antibody titers across six vaccine platforms of HIV envelope immunogens demonstrated a robust correlation between seminal plasma and rectal mucosal antibody titers in males but poor correlation between cervical and rectal antibody titers in females (28). The authors suggested that sex-specific features in the relationship between serum and genital/rectal mucosal antibody titers may affect the degree of protection (28). Emerging data from phase IIb trials of prophylactic administration of the broadly neutralizing antibody (bNAb) VRC01 provide some further insight into the function of humoral responses at mucosal sites (29). In these trials, one enrolling at-risk cisgender men and transgender individuals in Europe and the Americas and the other enrolling at-risk cisgender women in sub-Saharan Africa, VRC01 was not effective, but both trials showed a signal of preventive efficacy against viruses sensitive to neutralization by VRC01 that was linked to antibody concentration (29). In a separate analysis of the mucosal penetration of VRC01 in healthy volunteers, both rectal and vaginal explants demonstrated resistance to ex vivo challenge with sensitive strains of HIV (30). As strategies of combinations of antibodies for prevention with targeted modifications of the Fc region to enhance mucosal penetration and effector function are pursued, careful evaluation of the sex-specific accumulation and efficacy of these agents will be essential.

### Impact of local inflammation on HIV acquisition.

Both the efficacy of local immune responses and available target cells are directly affected by inflammation that may arise from either sexually transmitted infections (STIs) or as a result of the composition of the local microbiome. STIs including HSV-2, syphilis, gonorrhea, and chlamydia cause an increased risk of HIV acquisition during vaginal intercourse (31–35). A systematic review of the effect of herpes found that a relative risk of HIV acquisition was 2.7-fold higher with prevalent HSV-2 in the general population and 4.7-fold higher with incident infection, with no sex difference observed in the estimates (36). Likewise, an analysis of the impact of nonviral STIs demonstrated increased risk of HIV acquisition with coincident STI, although notably the data for males were sparse (37). Some of the enhanced risk may be attributable to behavioral patterns associated with STIs. Biological mechanisms include the influx of target cells to both the male and female genital tracts as a result of an STI (38–43); increased genital shedding of HIV driven by HSV-2 coinfection, which may directly influence HIV-1 acquisition (44); and disruption of the protective epithelial layer by genital ulceration in the setting of syphilis, chancroid, and HSV-2 infection (45, 46).

HIV seroconversion is also more likely when there is more inflammation, as defined by cervicovaginal levels of inflammatory cytokines such as MIP-1α, MIP-1β, and IP-10, which actively recruit target cells for HIV (47). In the absence of an STI, the specific composition of the vaginal microbiota, including when this shifts to a clinical diagnosis of bacterial vaginosis, is linked to increased risk of HIV acquisition (48–51). Inflammation driven by microbiota can activate Langerhans cells and CD4^+^ T cells, raising the risk of HIV acquisition (52, 53). Specific formulations of oral contraceptives have been linked to more-favorable vaginal microbial communities and to a lower frequency of STIs, suggesting that hormone modulation is a potential risk-modifying strategy (54, 55). The vaginal microbiome is also a critical consideration for topical pre-exposure prophylaxis, as certain species metabolize the antiviral drug tenofovir, lowering its preventive efficacy for HIV (56). Thus, the vaginal microbiome can confer risk, and understanding variations based on region, ethnicity, and local environment will be important to optimize prevention interventions (51, 57, 58). In parallel, the penile microbiome comprises specific microbial components that promote risk of acquisition, with the notable difference that medical male circumcision can significantly ameliorate, although not eliminate, the risk of seroconversion (22, 59, 60).

### Vertical transmission.

Intrauterine transmission is an emerging area of sex differential HIV transmission. In a recent single-site cohort study of infants with intrauterine acquisition of HIV, females outnumbered males 1.7:1, consistent with prior studies. This ratio contrasts with the sex ratios of HIV-exposed but uninfected infants and to the overall ratio of sex at birth in the study region (50.6% male) (61). Since the 1990s, multiple studies have assessed the risk of vertical transmission in the context of intrapartum antiretroviral therapy (ART), ART during pregnancy, and various approaches to infant treatment, with very early signals of an increased risk for female infants (62). In a large cohort study in Zimbabwe of 4,495 women living with HIV and their infants between 1997 and 2000, female infants were at greater risk of in utero acquisition (OR 1.53, 95% CI 1.23–1.91), despite 50.4% of all births being male (63, 64). An analysis of more than 2,000 women in Malawi in the 1990s reported higher rates of intrauterine acquisition in female infants (OR 1.4, 95% CI 0.2–2.2) and, notably, in 8 sets of sex-discordant twin pairs, 7 female infants and 1 male infant acquired HIV in utero (65). This finding is important, as it implies a selective pressure from the infant, given that the maternal environment of these twin pregnancies is identical. Separate work from Malawi in the early 2000s again reported a higher risk for female infants (OR 2.06, 95% CI 1.49–2.85), and this estimate was adjusted for maternal viral load, a strong independent predictor of transmission (66). Beyond the African context, the European Collaborative Study of vertical transmission noted that among infants delivered by elective cesarean section (effectively eliminating risk of intrapartum transmission), female infants are at higher risk (2.14, 95% CI 1.14–4.00) after adjustment for antenatal ART use and time period (67). An Italian registry had similar findings of lower risk for male infants (68). While modern ART has substantially reduced vertical transmission, the enhanced risk in female infants appears to persist. A prospective infant treatment trial screened 10,622 infants between 2015 and 2018, identified 42 with HIV within 96 hours of birth, and enrolled 40. Of the 40 infants enrolled in the trial, 78% were female (69). While there are multiple features to consider — including maternal ART, survival of male versus female infants independent of HIV risk, and differences in transmission risk across the timing of delivery — the weight of the collective data indicates that there is a higher risk of intrauterine transmission of HIV to female infants.

The mechanism of this differential risk is unclear; the twin data suggest that there are features of the infant that drive the difference. Of note, recent work has indicated that viruses recovered from female infants were more likely to be interferon resistant and have differences in replication capacity (61, 70). Production of type I interferon in response to TLR7 stimulation is a prominent feature of sex differential immune responses, as discussed below, and may contribute to this difference in early life.

## Pathogenesis

### Viral load.

Multiple studies have demonstrated that in the absence of HIV treatment, females have lower set point viral loads than males, although this difference attenuates with progression to advanced disease (71–79). In a study of individuals not on ART, females had less plasma virus associated with each HIV RNA^+^ CD4^+^ T cell in lymph node biopsies, suggesting that lower plasma viremia is associated with each HIV-infected cell in females (80). The lower systemic viral load is not protective, and males and females exhibit a similar time course of disease progression following seroconversion. This discordance meant that early on in the HIV epidemic, treatment guidelines based on viral load excluded women who were at risk for disease progression (79), highlighting the need for analysis of population variation for health policy–level decisions.

There are also important sex differences in the rates of the rare phenomenon of spontaneous control. In multiple large medical record database studies, rates of viremic and elite control are substantially higher in females, with the OR of female control ranging from 1.9 to 5 (81–84). Female participation in studies of elite controllers has not been representative; for example, an international cohort of 9,705 participants in a study that investigated the genetic determinants of HIV control was 82% male (85), leaving open questions about the effect of sex on this phenotype. Separate from spontaneous control is the phenomenon of posttreatment control, in which individuals who have been viremic are able to maintain viral suppression after a period of ART despite subsequent treatment discontinuation. The determinants of this type of control are under active investigation as a potential model of a functional cure. In one cohort of primary HIV infection, female sex was associated with a higher rate of posttreatment control (86). In other cohorts, there was not a clear signal for enrichment of control among females (87, 88), although the identification of individuals demonstrating posttreatment control was biased by the same factors that have led to the overrepresentation of males in other studies of HIV control described above. In a prospective trial assessing whether short-course ART in primary HIV infection leads to prolonged time to disease progression after ART interruption (89), female sex was a strong predictor of maintaining a viral load of fewer than 400 copies/mL for a longer period of time (90). However, the 40% of participants in the trial who were female were almost exclusively enrolled in African sites, and the contributions of the various geographic locations and HIV-1 virus clades cannot be completely separated from the contributions of the sex of the participants. In an analysis of ART discontinuation in more than 1,000 postpartum women treated during pregnancy as part of the PROMISE trial, 25% of the women remained virally suppressed (<400 copies/mL) at 12 weeks. This is a substantially higher level than the 6.4% of participants who maintained suppression at the same time point after treatment interruption in a comparator group of studies; notably, the comparator group was more than 90% male (91). Again, the effects of location, HIV virus clade, and pregnancy are difficult to disentangle from the effects of sex on the timing of viral rebound. Taken together, the data suggest a higher likelihood of spontaneous control in females, and there are suggestions of a higher likelihood of posttreatment control or significantly prolonged time to viral rebound in females.

### Innate and adaptive immune activation.

**Figure 1:**
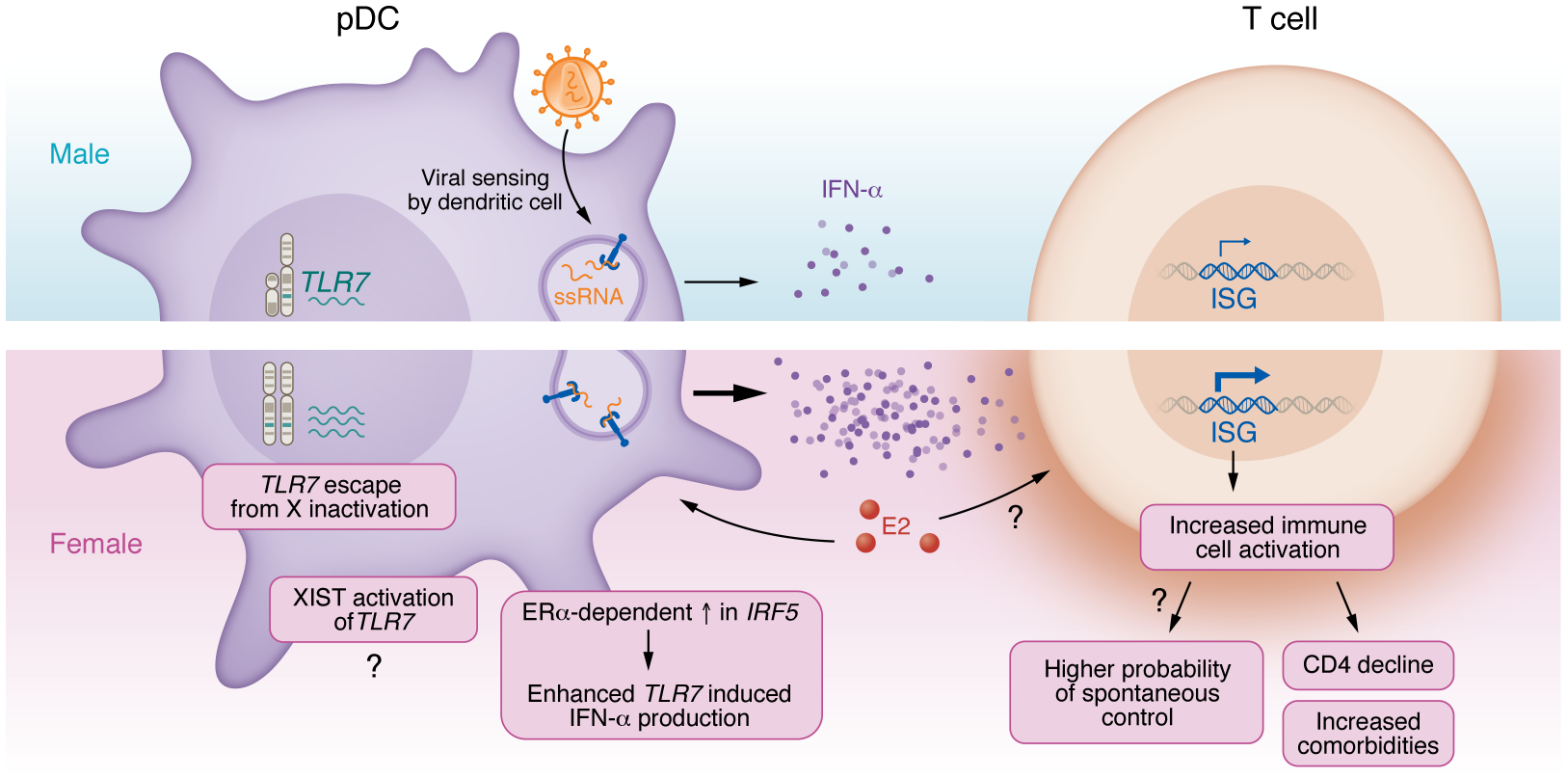
Multilevel effect of sex on HIV pathogenesis. *TLR7* escape from X inactivation in female plasmacytoid DCs (pDC) induces increased IFN-α levels. Increased IFN-α is in part a result of 17β-estradiol– (E2-) and ERα-dependent increases in *IRF5* expression. Expression of the long noncoding RNA XIST, which mediates epigenetic silencing of one X chromosome, also provides a source of TLR7 ligands that may enhance IFN-α. IFN-α promotes expression of interferon-stimulated genes (ISG) linked to increased immune cell activation. This enhanced response may contribute to higher frequency of controller phenotypes in females, but in chronic infection it drives CD4^+^ T cell decline and comorbidities.

A key driver of HIV pathogenesis is immune activation, with early studies demonstrating the association of T cell activation with progression to advanced disease (92, 93) (Figure 1). While females tend to have lower viral loads, the level of T cell activation for a given viral load is higher in females than in males (94). In untreated disease, type 1 interferon gene signatures were also higher in females, when controlled for viral load (95). Beyond HIV, females are generally described as having higher antiviral immune responses, a higher proportion of CD4^+^ T cells, increased production of IFN-α, and enhanced antibody production (4, 96, 97). Thus, one hypothesis is that a more robust response to HIV, as seen in higher production of IFN-α from plasmacytoid DCs (pDCs) after stimulation by HIV or other TLR7 ligands (94, 98, 99), may have two possible consequences: The first is a higher likelihood of virologic control as observed in the higher frequency of female spontaneous controllers discussed above. The second is a higher level of ongoing inflammation despite failure to control or eliminate the virus; this outcome would be linked to greater immune activation and risk of disease progression at a lower level of virus exposure. In studies assessing the rates of disease progression in males and females, lower viral load is not protective, with women progressing at similar rates despite lower median viral load levels (79); at least one study suggests that women progress at a faster rate (100). Higher levels of interferon-induced gene signatures in females may also be linked to the cell-intrinsic restriction of HIV replication and potentially lower per-cell production of HIV observed in lymph node CD4^+^ T cells in females (80). This has been described in macrophages, where female-derived cells had lower levels of HIV replication and higher levels of SAMHD1-based restriction (101). In recent work from murine model systems, isolated immune cells (macrophages, T and B cells) showed distinct patterns of interferon-stimulated gene transcription, notably with cells from female animals responding faster across all conditions (102). Taken together, the data suggest that a robust early antiviral response by females may be linked to lower viral loads, but at the cost of higher immune activation in chronic untreated HIV.

Emerging data about sex differences in intrauterine transmission again show links to interferon-based restriction, with viruses transmitted to females more likely to be interferon resistant (61, 70). Separate studies have confirmed that TLR7/TLR8 responses are lower in male infants (~2 months of age), confirming that differences in this axis are present even in early life (103). This immediately raises the question of which features of sex — genetic composition, sex steroid hormone exposure, epigenetic regulation — are underlying drivers of differences in immune response phenotype and viral restriction given the changes in these factors over a lifetime.

### Sex steroid hormones.

Sex steroid hormones and the expression and function of their receptors affect immune responses. In females, 17β-estradiol (E2), and progesterone concentrations fluctuate during the menstrual cycle and throughout life, while male androgen levels remain relatively consistent after puberty (104). In vitro studies showed that lower sex hormone concentrations, modeling the mid-proliferation hormone phase, are associated with higher levels of HIV transcription compared with the higher-concentration, midsecretory phase, suggesting that HIV replication is linked to hormone level (105).

Much of the literature on sex differences in HIV replication has focused on E2 and estrogen receptor α (ERα). ERα is activated upon E2 binding and is expressed in immune cells, and most studies have not demonstrated differences in expression at the transcriptional (106) or protein level (107) between males and females. ERα activation can induce nuclear localization and direct DNA binding at estrogen response elements (EREs) or indirect transcription effects via tethering transcription factors such as RUNX1, AP-1, and Sp1 (108–111). EREs have been found in the promoter region of many immune-related genes that affect activation (112), but it is unclear how the effect of E2 exposure intersects with direct immune-activating signals. Beyond the indirect effects E2 may have on host transcriptional machinery, in vitro studies demonstrated suppression of HIV replication by E2/ERα signaling (113). However, viral load levels in prepubertal females are lower than those in males even when E2 concentrations are similar between the sexes (114). The role of E2 in HIV transcriptional control in the context of ART is further discussed below in *Cure*.

Beyond these direct effects on viral dynamics, sex steroid hormones can also modulate immune pathways. Notably, the level of interferon regulatory factor 5 (IRF5), a downstream signaling component in the TLR7 response, is higher in pDCs from females and correlates with IFN-α production and with expression of ERα (115). TLR7 is also a canonical example of sex-specific genetic features, as discussed below.

### Genetics.

At the most basic level, sex differences in gene expression can arise from the chromosomal complement. Females have two copies of the X chromosome (XX), while males only have one (XY). One X chromosome in females undergoes X inactivation to normalize gene dosage between males and females, but X inactivation escape has emerged as a key contributor to sex differences (96, 116, 117). There are multiple immune active genes on the X chromosome, including TLR7, which has been shown to have dual expression in XX females and in XXY males (Klinefelter syndrome) in the immune system, with consequences for diseases including systemic lupus erythematosus (SLE) (118–121). Thus, females have higher TLR7 expression, and estrogen enhances the downstream signaling through IRF5. Further complicating this system is the recent identification that XIST, the long noncoding RNA that mediates X chromosome inactivation, acts as an endogenous TLR7 ligand, contributing to SLE pathogenesis (122, 123). Notably, a hypomorphic variant of *TLR7* has been described to have an effect on acute HIV viremia specifically in females, highlighting the sex-specific relationship between interferon and viral load (124). Taken together, the data indicate that gene dosage, hormone exposure, and epigenetic regulation all contribute to differences between males and females in the TLR7/interferon pathway.

Notwithstanding the importance of the sex chromosomes, the majority of sex-based gene expression variation in immune cells is derived from autosomal genes (106). There has been limited exploration of how sex-specific autosomal gene expression contributes to HIV outcomes. A recent study tested for sex chromosome and sex-stratified genomic markers in the largest GWAS of HIV set point viral load and spontaneous control (125). The analysis was limited by the relatively low representation of females in the cohort (<20%) but identified a gene-based association with set point viral load on chromosome 19 in males only and other gene variants with sex-discordant associations with set point viral load in potentially immune-active genes (125). Further work is needed to elucidate whether baseline or stimulated gene expression differences contribute to observed differences in immune response to HIV. In addition, another key gap in knowledge is the very limited body of work exploring immune cell function in transgender individuals with discordant sex chromosome complement and sex hormone exposure.

## Sex differences in the context of ART

### Treatment responses and comorbid conditions.

In general, both women and men achieve viral load suppression with ART, as predicted for medications that target viral proteins. As with many types of medications, for some ART agents there is a higher level of reported adverse effects in women and there are pharmacokinetic differences (126, 127). Analyses have historically been limited by low representation of women in clinical trials, which, although improving, still does not proportionally represent the epidemic, particularly regarding the inclusion of African women (15). For current ART, a major challenge is management of weight (128). The ADVANCE trial, a prospective randomized trial of three ART regimens, identified specific regimens as being linked to weight gain that is most pronounced among women (129, 130). The mechanisms by which these ART regimens promote weight gain are incompletely understood, and the intersection with sex may provide a key leverage point for understanding how these medications are affecting metabolism (131). Emerging work in preclinical models suggests that there may be an interaction among dolutegravir, estradiol, and mitochondrial function that may contribute to weight changes (132). Other possibilities — including effects of ART on the gut microbiome, which at baseline has sex-specific features (133) — are still under investigation.

Outside of the adverse effects of ART lies the residual inflammation from HIV even with near complete viral suppression. This inflammation is thought to be a driver of comorbid conditions and remains a key target of novel treatment strategies developed to ameliorate the effect of chronic HIV. Notably, HIV confers a proportionally greater increase in risk of cardiovascular and cerebrovascular disease in women as compared with men (134–137). These findings are consistent with sex-specific features of the burden of comorbid conditions, with changes also noted through reproductive aging in women (138–140). Some of this may reflect gender, with specific health-related behaviors including smoking that contribute to outcomes in women living with HIV. In the sub-Saharan African setting, male mortality exceeds female mortality, again thought to be driven in part by gendered differences in access to testing and care (141, 142). To optimize preventive health interventions across cis- and transgender individuals and in a variety of settings, more studies are needed to identify the HIV- and non-HIV-related drivers of inflammation and associations with comorbid illness and to separate gender- and sex-related mechanisms for disparities in outcomes.

### Cure.

**Figure 2:**
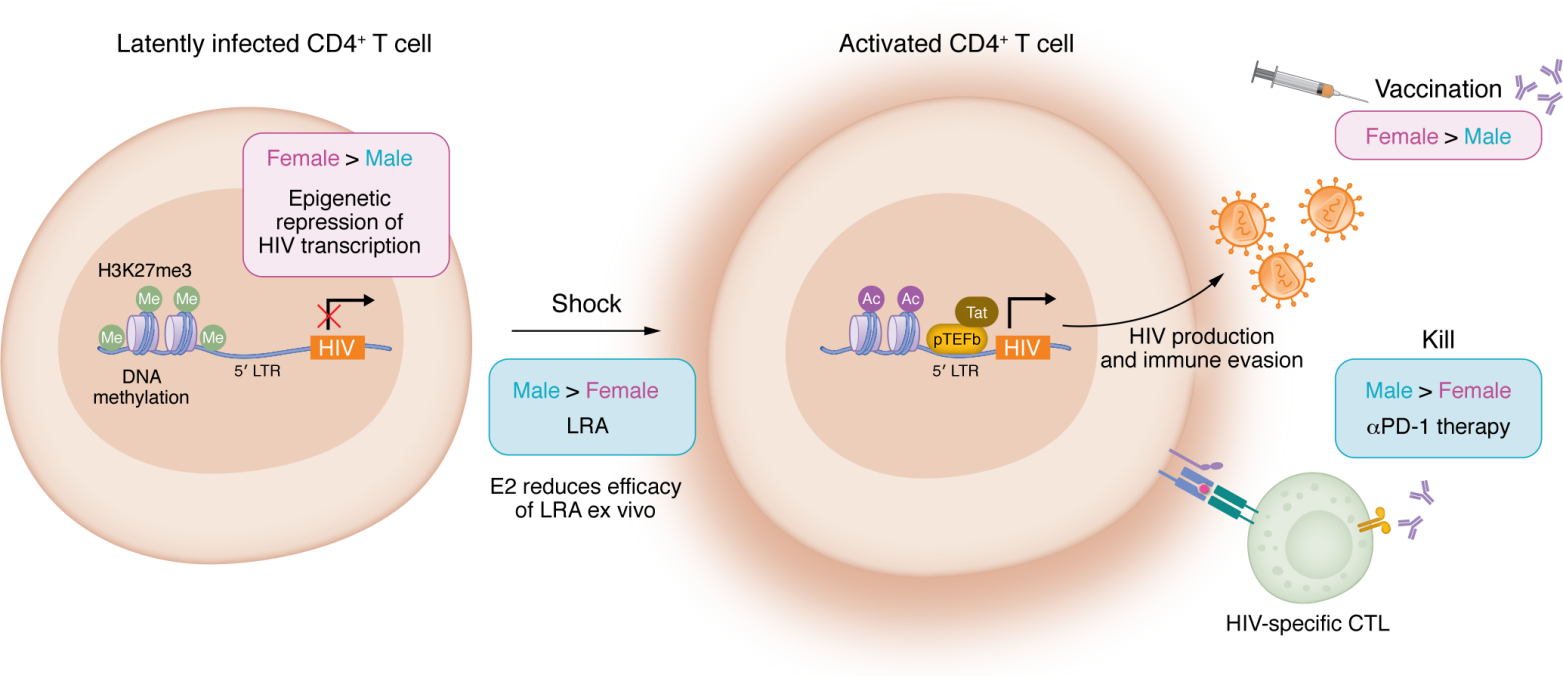
Sex differences in strategies for HIV cure. Female sex is associated with tighter control of latent HIV that may be a barrier to latency reversal. Mechanisms may include epigenetic repression and latency promotion via E2 signaling. Immune-enhancing strategies including checkpoint inhibition and vaccination may also have sex-differential efficacy. LRA, latency reversal agent; pTEFb, positive transcription elongation factor b; LTR, long terminal repeat; CTL, cytotoxic T lymphocyte.

Aside from eliminating residual inflammation, the other frontier of modern HIV clinical science is the effort to develop a curative intervention. Cure is variably defined as elimination of all replication-competent virus (eradication) or functional cure, whereby individuals no longer require daily ART to suppress HIV replication. The latter is a model of inducing a controller status and refers to the models of spontaneous control and posttreatment control described above with the notable influence of sex (143). Interestingly, all three individuals in the anecdotal reports characterized as having undergone spontaneous cure — i.e., no recovered replication-competent virus despite extensive sampling — were female (144–146). This, along with data suggesting that females are more likely to have a delayed rebound time after treatment interruption (discussed above) suggest that female sex may be associated with greater propensity to have sustained control (Figure 2).

Given the differences in set point viral load, studies directly explored whether there are differences in the low level of residual HIV expression observed under suppressive ART. In a cohort of matched reproductive-age men and women in the US, levels of HIV DNA were comparable, but levels of multiply spliced cell-associated HIV and low-level viremia by single-copy assay were lower in women (107). Lower levels of cell-associated HIV RNA in females were also observed in a retrospective analysis (147) and in a study of CMV/HIV coinfection (148). While some studies of peripheral blood mononuclear cells have suggested lower levels of total HIV DNA (149, 150), in the majority of studies, levels of HIV DNA (total and/or integrated) are comparable in men and women (107, 147, 148, 151, 152). This suggests tighter control of latent HIV expression in females as compared with males. It is unknown whether there is a difference in the replication competent reservoir; in one study, females had lower levels of ex vivo inducible HIV (152), but in another, there was no significant difference in measures of intact virus and outgrowth (151). These apparent differences in the stringency of latency maintenance are key to the feasibility of some curative strategies. Specifically, the approach of inducing HIV expression to allow identification and elimination of HIV reservoir–harboring cells, known as “shock and kill,” would be predicted to have a higher barrier in females (153). Alternatively, the strategy of “block and lock,” whereby integrated proviruses are maintained in a permanently silenced state of deep latency, might be easier to achieve in females (154). Given the challenges with achieving cure, even small differences in efficacy may be significant.

### Potential mechanisms of sex differences in HIV latency.

There is substantial interest in the potential mechanisms for sex-differential latency control. In an unbiased shRNA screen for host factors critical to maintenance of HIV latency, ERα emerged in three independent screens of a cell line model as a key latency regulator (155). This association was further tested using a primary cell model of latency and by assessing the effect of both estradiol and selective estrogen receptor antagonists designed to block or activate ERα. These studies consistently demonstrated that estrogen signaling blocked HIV latency reversal (155). In samples from PLWH, estradiol exposure blocked HIV RNA induction, and antagonists of ERα enhanced the latency reversal activity of other treatments, including the histone deacetylase inhibitor suberoylanilide hydroxamic acid (SAHA, also known as vorinostat) (107, 155). A clinical trial in postmenopausal women testing whether the selective estrogen receptor modulator tamoxifen could augment latency reversal with SAHA failed to show an increase in HIV RNA expression (156). This study was limited by the relatively poor latency reversal efficacy of SAHA and also by the low levels of detectable HIV RNA in trial participants, with substantially more participants having undetectable HIV RNA than in prior studies of male participants (156). In addition, this trial enrolled only postmenopausal women due to genotoxocity concerns around the use of SAHA. Subsequent work has highlighted that there is a higher level of HIV reactivation potential as women move through menopause with waning exposure to estradiol (157), suggesting that estradiol and tamoxifen are likely less impactful in postmenopausal women. Taken together, data support a role for estrogen and ERα in the regulation of HIV transcription, with a changing magnitude across the reproductive lifespan. The precise mechanism of this effect remains unknown.

Another potential mechanistic pathway for differences in HIV latency is sex specificity in epigenetic regulators. HIV latency induction and maintenance is partially mediated through epigenetic marks that suppress transcription through repressive nucleosome arrangements, DNA methylation, and histone methylation (158). Women have globally higher levels of DNA methylation in whole blood (159), and analyses of sex-biased gene expression across tissues suggest sex-differential epigenetic marks as a mechanism of differential gene expression (160). Again there is evidence of hormone modulation of these effects, with a smaller difference observed in postmenopausal women relative to men (161), highlighting the need to consider multiple features as potential mediators of differences.

A novel regulator of HIV infection susceptibility and reservoir maintenance lies in the metabolic state of the immune cell (162). HIV infection is less efficient in CD4^+^ T cells in glucose-deprived conditions, highlighting the importance of metabolic balance on HIV replication (163–165). Differences in metabolism between cisgender men and women are well appreciated, with women having higher body fat percentages than men and different adipose storage distribution, but there is limited exploration of the impact of sex on immunometabolism (166). The potential role of sex in metabolic control of immune cell function has not yet been explored in the context of HIV, but it may be identified as contributor to reservoir maintenance and anti-HIV responses.

### Curative therapies that may have sex-specific effects.

As highlighted in the previous section, sex differences in epigenetic regulation may lead to differences in therapeutic responses to latency reversal agents in this class of drugs. Another area of interest for latency reversal is TLR agonism, with a dual goal of boosting HIV expression and inducing immune responses to promote reservoir clearance (167, 168). Nonhuman primate studies had promising results, and several small clinical trials have explored the effect of TLR7 and TLR9 agonism on induction of HIV expression and reduction of reservoir size, with variable results (169–173). Representation of females was limited in these trials, insufficient to allow sex-specific analyses, but the abundant data on sex-specific features of TLR7 regulation and function suggest that this should be carefully considered.

Another potential source of variation is in strategies aimed at enhancing endogenous immune responses to more efficiently eliminate the reservoir. One approach is the use of immune checkpoint blockade therapies used in cancer therapy with the goal of reinvigorating the T cell response to eliminate HIV-infected cells (174, 175). In the prospective cohort of ART-suppressed participants exploring sex differences in reservoir activity, immunophenotyping showed lower expression of programmed cell death 1 (PD-1) on bulk CD4^+^ and CD8^+^ T cells from women as compared with men, although these measures do not provide information on antigen-specific responses (107). In cancer therapeutics, there are sex-specific patterns of response to checkpoint therapies across different tumors (176, 177). Taken together, “kill” strategies leveraging checkpoint blockade may be less effective in women. Conversely, other “kill” strategies may be more effective in women; therapeutic vaccines designed to augment and redirect the immune response to eliminate HIV reservoir cells are another potential immune-modulating strategy. A broad range of literature demonstrates generally more robust vaccine responses in females (reviewed in refs. 5, 178), arguing that these “kill” strategies may perform better in females.

## Opportunities

Sex differences in HIV acquisition and pathogenesis and their consequences for comorbidities and HIV cure efforts highlight multiple levels of the immune response to HIV (Table 2). They also highlight the risks of narrow representation in clinical trials and importance of testing interventions against population variation. Comparisons by sex remain a rich source of scientific discovery. Moving forward, further work is still necessary to clarify the role of sex steroid hormones and genetic and epigenetic controls in mediating differences in phenotype by sex. Work is needed to increase representation of cisgender women across the spectrum of clinical research and to investigate the unique setting of transgender individuals to allow the development of personalized care approaches. Deconvoluting the overall mechanisms of differences by sex in outcomes of HIV will be critical to developing prevention, treatment, and cure strategies that are efficacious across all people.

## Figures and Tables

**Table 1:**
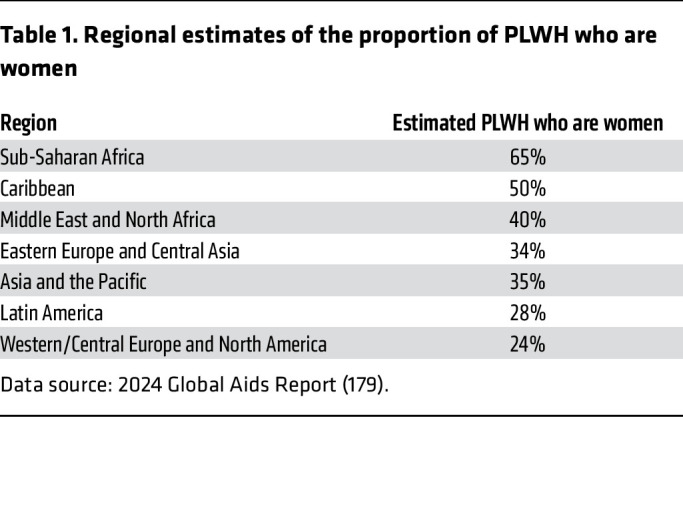
Regional estimates of the proportion of PLWH who are women

**Table 2:**
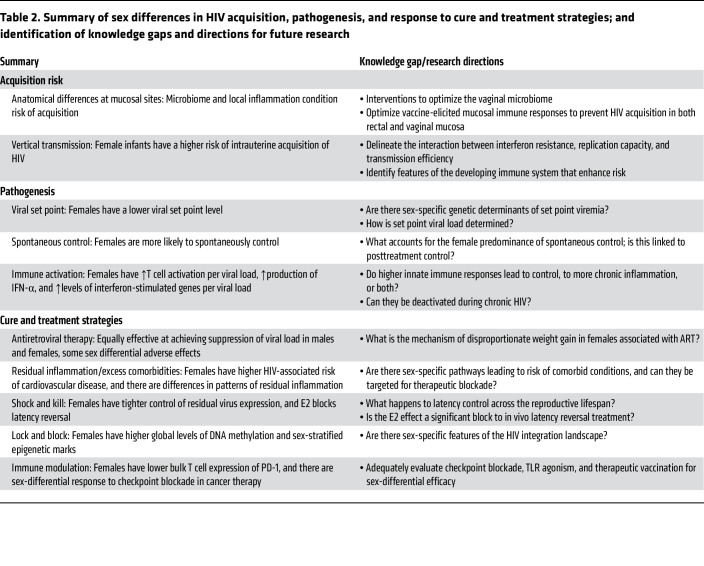
Summary of sex differences in HIV acquisition, pathogenesis, and response to cure and treatment strategies; and identification of knowledge gaps and directions for future research

## Version 1 09/17/2024

Electronic publication

## References

[1] Blankson JN, Siliciano RF (2008). Elite suppression of HIV-1 replication. Immunity.

[2] Deeks SG, Walker BD (2007). Human immunodeficiency virus controllers: mechanisms of durable virus control in the absence of antiretroviral therapy. Immunity.

[3] Olson AD (2014). Evaluation of rapid progressors in HIV infection as anextreme phenotype. J Acquir Immune Defic Syndr.

[4] Klein SL, Flanagan KL (2016). Sex differences in immune responses. Nat Rev Immunol.

[5] Klein SL (2010). The Xs and Y of immune responses to viral vaccines. Lancet Infect Dis.

[6] Markle JG, Fish EN (2014). SeXX matters in immunity. Trends Immunol.

[7] Mauvais-Jarvis F (2020). Sex and gender: modifiers of health, disease, and medicine. Lancet.

[8] Barr E (2024). Centring the health of women across the HIV research continuum. Lancet HIV.

[9] Frew PM (2016). Socioecological factors influencing women’s HIV risk in the United States: qualitative findings from the women’s HIV SeroIncidence study (HPTN 064). BMC Public Health.

[10] Centers for Disease Control (1981). Kaposi’s sarcoma and Pneumocystis pneumonia among homosexual men--New York City and California. MMWR Morb Mortal Wkly Rep.

[11] Centers for Disease Control (1981). Pneumocystis pneumonia--Los Angeles. MMWR Morb Mortal Wkly Rep.

[12] https://reliefweb.int/report/world/global-hiv-aids-statistics-fact-sheet-2023.

[13] Curno MJ (2016). A systematic review of the inclusion (or exclusion) of women in HIV research: from clinical studies of antiretrovirals and vaccines to cure strategies. J Acquir Immune Defic Syndr.

[14] Johnston RE, Heitzeg MM (2015). Sex, age, race and intervention type in clinical studies of HIV cure: a systematic review. AIDS Res Hum Retroviruses.

[15] Pepperrell T (2020). Phase 3 trials of new antiretrovirals are not representative of the global HIV epidemic. J Virus Erad.

[16] Hladik F, McElrath MJ (2008). Setting the stage: host invasion by HIV. Nat Rev Immunol.

[17] Patel P (2014). Estimating per-act HIV transmission risk: a systematic review. AIDS.

[18] Ackerley CG (2022). The rectal mucosal immune environment and HIV susceptibility among young men who have sex with men. Front Immunol.

[19] Boily M-C (2009). Heterosexual risk of HIV-1 infection per sexual act: systematic review and meta-analysis of observational studies. Lancet Infect Dis.

[20] Ballweber L (2011). Vaginal langerhans cells nonproductively transporting HIV-1 mediate infection of T cells. J Virol.

[21] Zhou Z (2011). HIV-1 efficient entry in inner foreskin is mediated by elevated CCL5/RANTES that recruits T cells and fuels conjugate formation with Langerhans cells. PLoS Pathog.

[22] Prodger JL (2022). How does voluntary medical male circumcision reduce HIV risk?. Curr HIV/AIDS Rep.

[23] Morrison CS (2015). Hormonal contraception and the risk of HIV acquisition: an individual participant data meta-analysis. PLoS Med.

[24] Polis CB (2016). Update on hormonal contraceptive methods and risk of HIV acquisition in women: a systematic review of epidemiological evidence, 2016. AIDS.

[25] Onono M (2020). Comparison of pregnancy incidence among African women in a randomized trial of intramuscular depot medroxyprogesterone acetate (DMPA-IM), a copper intrauterine device (IUDs) or a levonorgestrel (LNG) implant for contraception. Contracept X.

[26] Thomson KA (2018). Increased risk of HIV Acquisition among women throughout pregnancy and during the postpartum period: a prospective per-coital-act analysis among women with HIV-infected partners. J Infect Dis.

[27] Schuetz A (2023). Distinct mucosal and systemic immunological characteristics in transgender women potentially relating to HIV acquisition. JCI Insight.

[28] Seaton KE (2021). Meta-analysis of HIV-1 vaccine elicited mucosal antibodies in humans. NPJ Vaccines.

[29] Corey L (2021). Two randomized trials of neutralizing antibodies to prevent HIV-1 acquisition. N Engl J Med.

[30] Astronomo RD (2021). Rectal tissue and vaginal tissue from intravenous VRC01 recipients show protection against ex vivo HIV-1 challenge. J Clin Invest.

[31] Brown JM (2007). Incident and prevalent herpes simplex virus type 2 infection increases risk of HIV acquisition among women in Uganda and Zimbabwe. AIDS.

[32] Chun HM (2013). The role of sexually transmitted infections in HIV-1 progression: a comprehensive review of the literature. J Sex Transm Dis.

[33] Freeman EE (2006). Herpes simplex virus 2 infection increases HIV acquisition in men and women: systematic review and meta-analysis of longitudinal studies. AIDS.

[34] Masson L (2016). Inflammatory cytokine biomarkers to identify women with asymptomatic sexually transmitted infections and bacterial vaginosis who are at high risk of HIV infection. Sex Transm Infect.

[35] Masson L (2015). Genital inflammation and the risk of HIV acquisition in women. Clin Infect Dis.

[36] Looker KJ (2017). Effect of HSV-2 infection on subsequent HIV acquisition: an updated systematic review and meta-analysis. Lancet Infect Dis.

[37] Barker EK (2022). Risk of human immunodeficiency virus acquisition among high-risk heterosexuals with nonviral sexually transmitted infections: a systematic review and meta-analysis. Sex Transm Dis.

[38] Hladik F (1999). Coexpression of CCR5 and IL-2 in human genital but not blood T cells: implications for the ontogeny of the CCR5+ Th1 phenotype. J Immunol.

[39] Levine WC (1998). Increase in endocervical CD4 lymphocytes among women with nonulcerative sexually transmitted diseases. J Infect Dis.

[40] Patterson BK (1998). Repertoire of chemokine receptor expression in the female genital tract: implications for human immunodeficiency virus transmission. Am J Pathol.

[41] Sheffield JS (2007). Effect of genital ulcer disease on HIV-1 coreceptor expression in the female genital tract. J Infect Dis.

[42] Johnson KE (2011). Effects of HIV-1 and herpes simplex virus type 2 infection on lymphocyte and dendritic cell density in adult foreskins from Rakai, Uganda. J Infect Dis.

[43] Prodger JL (2012). Impact of asymptomatic Herpes simplex virus-2 infection on T cell phenotype and function in the foreskin. AIDS.

[44] Nagot N (2007). Reduction of HIV-1 RNA levels with therapy to suppress herpes simplex virus. N Engl J Med.

[45] Piot P, Laga M (1989). Genital ulcers, other sexually transmitted diseases, and the sexual transmission of HIV. BMJ.

[46] Weiler AM (2008). Genital ulcers facilitate rapid viral entry and dissemination following intravaginal inoculation with cell-associated simian immunodeficiency virus SIVmac239. J Virol.

[47] Masson L (2014). Defining genital tract cytokine signatures of sexually transmitted infections and bacterial vaginosis in women at high risk of HIV infection: a cross-sectional study. Sex Transm Infect.

[48] Anahtar MN (2015). Cervicovaginal bacteria are a major modulator of host inflammatory responses in the female genital tract. Immunity.

[49] McClelland RS (2018). Evaluation of the association between the concentrations of key vaginal bacteria and the increased risk of HIV acquisition in African women from five cohorts: a nested case-control study. Lancet Infect Dis.

[50] Mitchell C (2015). Hydrogen peroxide-producing lactobacilli are associated with lower levels of vaginal interleukin-1β, independent of bacterial vaginosis. Sex Transm Dis.

[51] Ravel J (2011). Vaginal microbiome of reproductive-age women. Proc Natl Acad Sci U S A.

[52] Gosmann C (2017). Lactobacillus-deficient cervicovaginal bacterial communities are associated with increased HIV acquisition in young South African women. Immunity.

[53] van Teijlingen NH (2023). Immune activation of vaginal human Langerhans cells increases susceptibility to HIV-1 infection. Sci Rep.

[54] Balle C (2021). Hormonal contraception and risk of STIs and bacterial vaginosis in South African adolescents: secondary analysis of a randomised trial. Sex Transm Infect.

[55] Balle C (2020). Hormonal contraception alters vaginal microbiota and cytokines in South African adolescents in a randomized trial. Nat Commun.

[56] Klatt NR (2017). Vaginal bacteria modify HIV tenofovir microbicide efficacy in African women. Science.

[57] Gupta VK (2017). Geography, ethnicity or subsistence-specific variations in human microbiome composition and diversity. Front Microbiol.

[58] Roachford OSE (2022). Insights into the vaginal microbiome in a diverse group of women of African, Asian and European ancestries. PeerJ.

[59] Kaul R (2022). The penis, the vagina and HIV risk: key differences (aside from the obvious). Viruses.

[60] Prodger JL (2021). Penile bacteria associated with HIV seroconversion, inflammation, and immune cells. JCI Insight.

[61] Adland E (2020). Sex-specific innate immune selection of HIV-1 in utero is associated with increased female susceptibility to infection. Nat Commun.

[62] Temmerman M (1995). Risk factors for mother-to-child transmission of human immunodeficiency virus-1 infection. Am J Obstet Gynecol.

[63] Marinda E (2007). Child mortality according to maternal and infant HIV status in Zimbabwe. Pediatr Infect Dis J.

[64] Piwoz EG (2006). Effects of infant sex on mother-to-child transmission of HIV-1 according to timing of infection in Zimbabwe. AIDS.

[65] Biggar RJ (2006). Higher in utero and perinatal HIV infection risk in girls than boys. J Acquir Immune Defic Syndr.

[66] Taha TE (2005). Gender differences in perinatal HIV acquisition among African infants. Pediatrics.

[67] Thorne C (2004). Are girls more at risk of intrauterine-acquired HIV infection than boys?. AIDS.

[68] Galli L (2005). Lower mother-to-child HIV-1 transmission in boys is independent of type of delivery and antiretroviral prophylaxis: the Italian Register for HIV Infection in Children. J Acquir Immune Defic Syndr.

[69] Maswabi K (2021). Safety and efficacy of starting antiretroviral therapy in the first week of life. Clin Infect Dis.

[70] Bengu N Sustained aviremia despite anti-retroviral therapy non-adherence in male children after in utero HIV transmission. Nat Med.

[71] Anastos K (2000). Association of race and gender with HIV-1 RNA levels and immunologic progression. J Acquir Immune Defic Syndr.

[72] Evans JS (1997). Serum levels of virus burden in early-stage human immunodeficiency virus type 1 disease in women. J Infect Dis.

[73] Farzadegan H (1998). Sex differences in HIV-1 viral load and progression to AIDS. Lancet.

[74] Gandhi M (2002). Does patient sex affect human immunodeficiency virus levels?. Clin Infect Dis.

[75] Katzenstein DA (1996). The relation of virologic and immunologic markers to clinical outcomes after nucleoside therapy in HIV-infected adults with 200 to 500 CD4 cells per cubic millimeter. AIDS Clinical Trials Group Study 175 Virology Study Team. N Engl J Med.

[76] Lyles CM (1999). Longitudinal human immunodeficiency virus type 1 load in the italian seroconversion study: correlates and temporal trends of virus load. J Infect Dis.

[77] Napravnik S (2002). Gender difference in HIV RNA levels: a meta-analysis of published studies. J Acquir Immune Defic Syndr.

[78] Sterling TR (1999). Sex differences in longitudinal human immunodeficiency virus type 1 RNA levels among seroconverters. J Infect Dis.

[79] Sterling TR (2001). Initial plasma HIV-1 RNA levels and progression to AIDS in women and men. N Engl J Med.

[80] Meditz AL (2014). CCR5 expression is reduced in lymph nodes of HIV type 1-infected women, compared with men, but does not mediate sex-based differences in viral loads. J Infect Dis.

[81] Crowell TA (2015). Hospitalization rates and reasons among HIV elite controllers and persons with medically controlled HIV infection. J Infect Dis.

[82] Madec Y (2005). Spontaneous control of viral load and CD4 cell count progression among HIV-1 seroconverters. AIDS.

[83] Price MA (2019). Control of the HIV-1 load varies by viral subtype in a large cohort of African adults with incident HIV-1 infection. J Infect Dis.

[84] Yang OO (2017). Demographics and natural history of HIV-1-infected spontaneous controllers of viremia. AIDS.

[85] International HIVCS (2010). The major genetic determinants of HIV-1 control affect HLA class I peptide presentation. Science.

[86] Goujard C (2012). HIV-1 control after transient antiretroviral treatment initiated in primary infection: role of patient characteristics and effect of therapy. Antivir Ther.

[87] Namazi G (2018). The control of HIV after antiretroviral medication pause (CHAMP) study: posttreatment controllers identified from 14 clinical studies. J Infect Dis.

[88] Saez-Cirion A (2013). Post-treatment HIV-1 controllers with a long-term virological remission after the interruption of early initiated antiretroviral therapy ANRS VISCONTI Study. PLoS Pathog.

[89] Investigators ST (2013). Short-course antiretroviral therapy in primary HIV infection. N Engl J Med.

[90] Stohr W (2013). Duration of HIV-1 viral suppression on cessation of antiretroviral therapy in primary infection correlates with time on therapy. PLoS One.

[91] Le CN (2019). Time to viral rebound and safety after antiretroviral treatment interruption in postpartum women compared with men. AIDS.

[92] Giorgi JV (1999). Shorter survival in advanced human immunodeficiency virus type 1 infection is more closely associated with T lymphocyte activation than with plasma virus burden or virus chemokine coreceptor usage. J Infect Dis.

[93] Deeks SG (2004). Immune activation set point during early HIV infection predicts subsequent CD4^+^ T-cell changes independent of viral load. Blood.

[94] Meier A (2009). Sex differences in the Toll-like receptor-mediated response of plasmacytoid dendritic cells to HIV-1. Nat Med.

[95] Chang JJ (2013). Higher expression of several interferon-stimulated genes in HIV-1-infected females after adjusting for the level of viral replication. J Infect Dis.

[96] Forsyth KS (2024). The conneXion between sex and immune responses. Nat Rev Immunol.

[97] Bongen E (2019). Sex differences in the blood transcriptome identify robust changes in immune cell proportions with aging and influenza infection. Cell Rep.

[98] Berghöfer B (2006). TLR7 ligands induce higher IFN-α production in females. J Immunol.

[99] Ziegler SM (2017). Human pDCs display sex-specific differences in type I interferon subtypes and interferon α/β receptor expression. Eur J Immunol.

[100] Parsa N (2020). The rapid CD4+ T-lymphocyte decline and human immunodeficiency virus progression in females compared to males. Sci Rep.

[101] Szaniawski MA (2019). Sex influences SAMHD1 activity and susceptibility to human immunodeficiency virus-1 in primary human macrophages. J Infect Dis.

[102] Gal-Oz ST (2024). Microheterogeneity in the kinetics and sex-specific response to type I IFN. J Immunol.

[103] Wang JP (2012). Plasmacytoid dendritic cell interferon-α production to R-848 stimulation is decreased in male infants. BMC Immunol.

[104] Hoffmann JP (2023). Sex hormone signaling and regulation of immune function. Immunity.

[105] Asin SN (2008). Estradiol and progesterone regulate HIV type 1 replication in peripheral blood cells. AIDS Res Hum Retroviruses.

[106] Schmiedel BJ (2018). Impact of genetic polymorphisms on human immune cell gene expression. Cell.

[107] Scully EP (2019). Sex-based differences in human immunodeficiency virus type 1 reservoir activity and residual immune activation. J Infect Dis.

[108] Cheung E (2005). Altered pharmacology and distinct coactivator usage for estrogen receptor-dependent transcription through activating protein-1. Proc Natl Acad Sci U S A.

[109] Cheung E, Kraus WL (2010). Genomic analyses of hormone signaling and gene regulation. Annu Rev Physiol.

[110] Porter W (1997). Functional synergy between the transcription factor Sp1 and the estrogen receptor. Mol Endocrinol.

[111] Stender JD (2010). Genome-wide analysis of estrogen receptor alpha DNA binding and tethering mechanisms identifies Runx1 as a novel tethering factor in receptor-mediated transcriptional activation. Mol Cell Biol.

[112] Hewagama A (2013). Overexpression of X-linked genes in T cells from women with lupus. J Autoimmun.

[113] Szotek EL (2013). 17β-estradiol inhibits HIV-1 by inducing a complex formation between β-catenin and estrogen receptor α on the HIV promoter to suppress HIV transcription. Virology.

[114] Ruel TD (2011). Sex differences in HIV RNA level and CD4 cell percentage during childhood. Clin Infect Dis.

[115] Griesbeck M (2015). Sex differences in plasmacytoid dendritic cell levels of IRF5 drive higher IFN-α production in women. J Immunol.

[116] Dunford A (2017). Tumor-suppressor genes that escape from X-inactivation contribute to cancer sex bias. Nat Genet.

[117] Libert C (2010). The X chromosome in immune functions: when a chromosome makes the difference. Nat Rev Immunol.

[118] Hagen SH (2020). Heterogeneous escape from X Chromosome inactivation results in sex differences in type I IFN responses at the single human pDC level. Cell Rep.

[119] Souyris M (2018). *TLR7* escapes X chromosome inactivation in immune cells. Sci Immunol.

[120] Souyris M (2019). Female predisposition to TLR7-driven autoimmunity: gene dosage and the escape from X chromosome inactivation. Semin Immunopathol.

[121] Laffont S (2014). X-Chromosome complement and estrogen receptor signaling independently contribute to the enhanced TLR7-mediated IFN-α production of plasmacytoid dendritic cells from women. J Immunol.

[122] Crawford JD (2023). , Thomas MA, et al. The XIST lncRNA is a sex-specific reservoir of TLR7 ligands in SLE. JCI Insight.

[123] Dou DR (2024). Xist ribonucleoproteins promote female sex-biased autoimmunity. Cell.

[124] Azar P (2020). TLR7 dosage polymorphism shapes interferogenesis and HIV-1 acute viremia in women. JCI Insight.

[125] Vergara C (2023). Multiancestry sex-stratified genomic associations with HIV viral load and controller status from the ICGH. JCI Insight.

[126] Ofotokun I, Pomeroy C (2003). Sex differences in adverse reactions to antiretroviral drugs. Top HIV Med.

[127] Umeh OC, Currier JS (2006). Sex differences in pharmacokinetics and toxicity of antiretroviral therapy. Expert Opin Drug Metab Toxicol.

[128] Sax PE (2020). Weight gain following initiation of antiretroviral therapy: risk factors in randomized comparative clinical trials. Clin Infect Dis.

[129] Sokhela S (2024). Final 192-week efficacy and safety results of the ADVANCE trial, comparing 3 first-line antiretroviral regimens. Open Forum Infect Dis.

[130] Venter WDF (2019). Dolutegravir plus two different prodrugs of tenofovir to treat HIV. N Engl J Med.

[131] Chandiwana NC (2023). Weight gain after HIV therapy initiation: pathophysiology and implications. J Clin Endocrinol Metab.

[132] Jung I (2022). Dolutegravir suppresses thermogenesis via disrupting uncoupling protein 1 expression and mitochondrial function in brown/beige adipocytes in preclinical models. J Infect Dis.

[133] Markle JG (2013). Sex differences in the gut microbiome drive hormone-dependent regulation of autoimmunity. Science.

[134] Chow FC (2012). Comparison of ischemic stroke incidence in HIV-infected and non-HIV-infected patients in a US health care system. J Acquir Immune Defic Syndr.

[135] Chow FC (2018). Elevated ischemic stroke risk among women living with HIV infection. AIDS.

[136] Raghavan A (2017). Sex differences in select non-communicable HIV-associated comorbidities: exploring the role of systemic immune activation/inflammation. Curr HIV/AIDS Rep.

[137] Triant VA (2007). Increased acute myocardial infarction rates and cardiovascular risk factors among patients with human immunodeficiency virus disease. J Clin Endocrinol Metab.

[138] Collins LF (2023). The effect of menopausal status, age, and human immunodeficiency virus (HIV) on non-AIDS comorbidity burden among US women. Clin Infect Dis.

[139] Collins LF (2021). The prevalence and burden of non-AIDS comorbidities among women living with or at risk for human immunodeficiency virus infection in the United States. Clin Infect Dis.

[140] Pond RA (2021). Sex differences in non-AIDS comorbidities among people with human immunodeficiency virus. Open Forum Infect Dis.

[141] Dovel K (2015). Men’s heightened risk of AIDS-related death: the legacy of gendered HIV testing and treatment strategies. AIDS.

[142] Kerkhoff AD (2020). Mortality estimates by age and sex among persons living with HIV after ART initiation in Zambia using electronic medical records supplemented with tracing a sample of lost patients: A cohort study. PLoS Med.

[143] Li JZ, Blankson JN (2021). How elite controllers and posttreatment controllers inform our search for an HIV-1 cure. J Clin Invest.

[144] Jiang C (2020). Distinct viral reservoirs in individuals with spontaneous control of HIV-1. Nature.

[145] Turk G (2022). A possible sterilizing cure of HIV-1 infection without stem cell transplantation. Ann Intern Med.

[146] Uruena A (2021). Prolonged posttreatment virologic control and complete seroreversion after advanced human immunodeficiency virus-1 infection. Open Forum Infect Dis.

[147] Gandhi RT (2017). Levels of HIV-1 persistence on antiretroviral therapy are not associated with markers of inflammation or activation. PLoS Pathog.

[148] Gianella S (2020). Sex differences in CMV replication and HIV persistence during suppressive ART. Open Forum Infect Dis.

[149] Cuzin L (2015). Levels of intracellular HIV-DNA in patients with suppressive antiretroviral therapy. AIDS.

[150] Fourati S (2014). Factors associated with a low HIV reservoir in patients with prolonged suppressive antiretroviral therapy. J Antimicrob Chemother.

[151] Falcinelli SD (2020). Impact of biological sex on immune activation and frequency of the latent HIV reservoir during suppressive antiretroviral therapy. J Infect Dis.

[152] Prodger JL (2020). Reduced HIV-1 latent reservoir outgrowth and distinct immune correlates among women in Rakai, Uganda. JCI Insight.

[153] Deeks SG (2012). HIV: Shock and kill. Nature.

[154] Elsheikh MM (2019). Deep latency: a new insight into a functional HIV cure. EBioMedicine.

[155] Das B (2018). Estrogen receptor-1 is a key regulator of HIV-1 latency that imparts gender-specific restrictions on the latent reservoir. Proc Natl Acad Sci U S A.

[156] Scully EP (2022). Impact of tamoxifen on vorinostat-induced human immunodeficiency virus expression in women on antiretroviral therapy: AIDS Clinical Trials Group A5366, the MOXIE trial. Clin Infect Dis.

[157] Gianella S (2022). Sex differences in human immunodeficiency virus persistence and reservoir size during aging. Clin Infect Dis.

[158] Verdikt R (2021). Epigenetic mechanisms of HIV-1 persistence. Vaccines (Basel).

[159] Grant OA (2022). Characterising sex differences of autosomal DNA methylation in whole blood using the Illumina EPIC array. Clin Epigenetics.

[160] Oliva M (2020). The impact of sex on gene expression across human tissues. Science.

[161] Jansen R (2014). Sex differences in the human peripheral blood transcriptome. BMC Genomics.

[162] Sáez-Cirión A, Sereti I (2021). Immunometabolism and HIV-1 pathogenesis: food for thought. Nat Rev Immunol.

[163] Clerc I (2019). Entry of glucose- and glutamine-derived carbons into the citric acid cycle supports early steps of HIV-1 infection in CD4 T cells. Nat Metab.

[164] Hegedus A (2014). HIV-1 pathogenicity and virion production are dependent on the metabolic phenotype of activated CD4+ T cells. Retrovirology.

[165] Valle-Casuso JC (2019). Cellular metabolism is a major determinant of HIV-1 reservoir seeding in CD4^+^ T cells and offers an opportunity to tackle infection. Cell Metab.

[166] Manuel RSJ, Liang Y (2021). Sexual dimorphism in immunometabolism and autoimmunity: impact on personalized medicine. Autoimmun Rev.

[167] Macedo AB (2018). Dual TLR2 and TLR7 agonists as HIV latency-reversing agents. JCI Insight.

[168] Martinsen JT (2020). The use of toll-like receptor agonists in HIV-1 cure strategies. Front Immunol.

[169] Krarup AR (2018). The TLR9 agonist MGN1703 triggers a potent type I interferon response in the sigmoid colon. Mucosal Immunol.

[170] Riddler SA (2020). Vesatolimod, a toll-like receptor 7 agonist, induces immune activation in virally suppressed adults living with human immunodeficiency virus-1. Clin Infect Dis.

[171] SenGupta D (2021). The TLR7 agonist vesatolimod induced a modest delay in viral rebound in HIV controllers after cessation of antiretroviral therapy. Sci Transl Med.

[172] Vibholm L (2017). Short-course Toll-like receptor 9 agonist treatment impacts innate immunity and plasma viremia in individuals with human immunodeficiency virus infection. Clin Infect Dis.

[173] Vibholm LK (2019). Effects of 24-week Toll-like receptor 9 agonist treatment in HIV type 1+ individuals. AIDS.

[174] Gubser C (2022). Immune checkpoint blockade in HIV. EBioMedicine.

[175] Gay CL (2017). Clinical trial of the anti-PD-L1 antibody BMS-936559 in HIV-1 infected participants on suppressive antiretroviral therapy. J Infect Dis.

[176] Conforti F (2021). Sex-based differences in response to anti-PD-1 or PD-L1 treatment in patients with non-small-cell lung cancer expressing high PD-L1 levels. A systematic review and meta-analysis of randomized clinical trials. ESMO Open.

[177] Jang SR (2021). Association between sex and immune checkpoint inhibitor outcomes for patients with melanoma. JAMA Netw Open.

[178] Fink AL, Klein SL (2015). Sex and gender impact immune responses to vaccines among the elderly. Physiology (Bethesda).

[179] https://www.unaids.org/en/resources/documents/2024/global-aids-update-2024.

